# Seladelpar combined with complementary therapies improves fibrosis, inflammation, and liver injury in a mouse model of nonalcoholic steatohepatitis

**DOI:** 10.1152/ajpgi.00158.2023

**Published:** 2023-11-28

**Authors:** Yun-Jung Choi, Jeff D. Johnson, Jin-Ju Lee, Jiangao Song, Marcy Matthews, Marc K. Hellerstein, Charles A. McWherter

**Affiliations:** ^1^CymaBay Therapeutics, Inc., Fremont, California, United States; ^2^Department of Nutritional Sciences and Toxicology, University of California, Berkeley, Berkeley, California, United States

**Keywords:** combination therapies, DIO-NASH, GLP-1 receptor agonist, liver fibrosis, PPARδ

## Abstract

Seladelpar, a selective peroxisome proliferator-activated receptor δ (PPARδ) agonist, improves markers of hepatic injury in human liver diseases, but histological improvement of nonalcoholic steatohepatitis (NASH) and liver fibrosis has been challenging with any single agent. To discover how complementary agents could work with seladelpar to achieve optimal outcomes, this study evaluated a variety of therapeutics (alone and in combination) in a mouse model of NASH. Mice on a high-fat amylin liver NASH (AMLN) diet were treated for 12 wk with seladelpar, GLP-1-R (glucagon-like peptide-1 receptor) agonist liraglutide, apoptosis signal-regulating kinase 1 (ASK1) inhibitor selonsertib, farnesoid X receptor (FXR) agonist obeticholic acid, and with seladelpar in combination with liraglutide or selonsertib. Seladelpar treatment markedly improved plasma markers of liver function. Seladelpar alone or in combination resulted in stark reductions in liver fibrosis (hydroxyproline, new collagen synthesis rate, mRNA indices of fibrosis, and fibrosis staining) compared with vehicle and the other single agents. Robust reductions in liver steatosis were also observed. Seladelpar produced a reorganization of metabolic gene expression, particularly for those genes promoting peroxisomal and mitochondrial lipid oxidation. In summary, substantial improvements in NASH and NASH-induced fibrosis were observed with seladelpar alone and in combination with liraglutide in this model. Broad gene expression analysis suggests seladelpar should be effective in concert with diverse mechanisms of action.

**NEW & NOTEWORTHY** NASH is a chronic, progressive, and increasingly problematic liver disease that has been resistant to treatment with individual therapeutics. In this study using a diet-induced mouse model of NASH, we found that the PPARδ agonist seladelpar reduced fibrosis and NASH pathology alone and in combinations with a GLP-1-R agonist (liraglutide) or an ASK1 inhibitor (selonsertib). Liver transcriptome analysis comparing each agent and coadministration suggests seladelpar should be effective in combination with a variety of therapeutics.

## Graphical abstract

**Figure:**
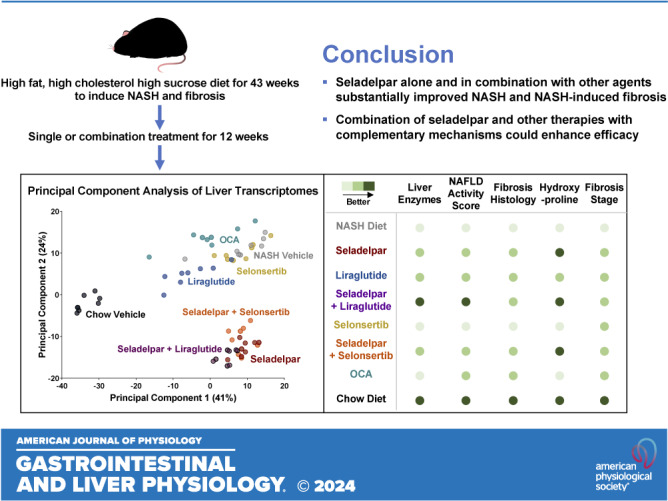

## INTRODUCTION

Nonalcoholic steatohepatitis (NASH) is a progressive chronic liver disease with histopathological features of excessive accumulation of hepatic fat and hepatocellular injury manifesting as ballooning or degeneration of hepatocytes, lobular inflammation, and apoptotic bodies. NASH presents a considerable risk of progression to liver cirrhosis and hepatocellular carcinoma (HCC). Liver fibrosis is a hallmark of NASH, and the degree of fibrosis is used to determine severity of the disease (1). In the absence of approved therapies, NASH is the most rapidly rising driver of liver transplantation regardless of HCC status in the United States (2).

Seladelpar, a selective and potent peroxisome proliferator-activated receptor δ (PPARδ) agonist, has demonstrated hepatoprotective effects in clinical studies (3–5) but, like other single agents, may need to be combined with treatments that use complementary mechanisms for more effective resolution of NASH and fibrosis. Unique among the PPAR family of transcription factors, PPARδ is ubiquitously expressed and thus may provide beneficial effects across multiple tissues. Studies with tissue-selective overexpression, tissue-specific knockouts, and selective PPARδ agonists indicate that this transcription factor exerts hepatic and peripheral effects through regulation of lipid metabolism, energy expenditure, and by reducing hepatic inflammation and fibrosis (6–10). For example, seladelpar leads to improvement in dyslipidemia, inflammation, fibrosis, and NASH pathology in a diabetic obese mouse model (11).

Therapeutics that may have orthogonal mechanisms of activity against NASH represent opportunities to explore complementarity for fibrosis improvement. Liraglutide, a GLP-1 receptor agonist (GLP-1-RA), has been approved for treatment of type 2 diabetes, obesity, and chronic weight management. In a placebo-controlled phase 2 study of liraglutide in patients with NASH, a significantly greater proportion of patients treated with liraglutide achieved resolution of NASH with no worsening in fibrosis compared with placebo group. However, liraglutide did not show improvement in fibrosis (12). Selonsertib, an apoptosis signal-regulating kinase 1 (ASK1) inhibitor, acts via blockade of the MAP kinase pathway, limiting the activity of downstream kinases of the P38 and JNK families, which mediate many of the effects of inflammation, endoplasmic reticulum (ER) and oxidative stress, and fibrosis in multiple tissues, including liver. Selonsertib has been the subject of clinical trials for NASH, but these have not demonstrated efficacy of selonsertib as a single agent (13, 14).

NASH has a multifaceted etiology, but the relative contribution of the elements of its progression is not well understood. Risk factors for NASH include components of metabolic syndrome: obesity, diabetes, and dyslipidemia. Despite high unmet clinical need and a plethora of efforts to develop a therapy for NASH, improvement of fibrosis and effective resolution of NASH is challenging and combining therapies with different complementary mechanisms of action may achieve better outcomes (15, 16). Here, we evaluated two drug combinations of seladelpar with liraglutide or selonsertib to further limit NASH pathology in a biopsy-confirmed, diet-induced obese mouse model of NASH. We also included the Farnesoid X receptor (FXR) agonist obeticholic acid (OCA) as a comparator. We examined the effect of each of the compounds and combinations on multiple read-outs, including liver biochemistry, liver histology and immunohistochemistry, gene expression, new collagen synthesis rate, and expressed genome patterns to map how each agent’s effects may interplay to support hepatocyte health and improve NASH pathologies.

## METHODS

### Animals, Diets, and Baseline Liver Biopsy

To obtain diet-induced obese NASH mice (DIO-NASH), male C57BL/6JRj mice aged 5 wk old were fed an amylin diet [AMLN, containing 40% fat (18% *trans*-fat), 40% carbohydrate (20% fructose), and 2% cholesterol; D09100310, Research Diets] (17) ad libitum for 43 wk to induce advanced histopathological features of NASH. A baseline liver biopsy was performed 3 wk before the drug treatment period as described previously (18). Only those mice (DIO-NASH cohort) with histologically confirmed steatosis ≥ 2 by hematoxylin-eosin stain (H&E) and fibrosis stage ≥ 1 by Picro-Sirius Red (PSR) (19) were stratified and randomly assigned to the treatment groups using a biopsy-based evaluation of liver type 1 collagen α1 (Col1a1) levels. Age-matched normal Chow mice were fed a Chow diet (Altromin 1324, Brogaarden, Denmark) for 43 wk. Throughout the study, the treatment group allocation was blinded to individuals who performed the study. Body weights were measured daily throughout the treatment period and food intake was assessed daily over the first 2 wk and then weekly over the next 10 wk. The AMLN diet was continued during the treatment period for a total of 55 wk. The overall study design is outlined in Supplemental Fig. S1. All experiments were approved by The Animal Experimentation Council, Danish Veterinary and Food Administration (license no. 2013-15-2934-00784) and the Gubra ethics committee. Animal experiments were conducted at Gubra (Hørsholm, Denmark) in compliance with internationally accepted principles for the care and use of laboratory animals.

### Drug Treatment

Mice were treated daily for 12 wk with vehicle (once a day, orally), seladelpar (10 mg/kg, once a day, orally) (11), liraglutide (0.2 mg/kg, twice a day, subcutaneous) (20), selonsertib (30 mg/kg, twice a day, orally) (21), obeticholic acid (OCA, 30 mg/kg, once a day, orally) (20), alone or in combinations of seladelpar with liraglutide or selonsertib (*n* = 12 mice/group). Carboxymethyl cellulose (0.5%) was used as an oral dosing vehicle and phosphate-buffered saline with 0.1% bovine serum albumin was used as a vehicle for liraglutide subcutaneous dosing. Chow-fed mice dosed with oral vehicle (once a day, orally) served as a normal, non-NASH comparison group (Chow Vehicle, *n* = 10).

### Plasma and Liver Biochemistry

Levels of plasma triglyceride (TG), total cholesterol (TC), alanine aminotransferase (ALT), aspartate aminotransferase (AST), and liver TG and TC were measured in a single run from blood and liver tissue taken at euthanasia as described previously (18). For liver hydroxyproline measurement, ∼100 mg of each liver sample was homogenized and hydrolyzed in 6 M HCl at 95°C for 16–20 h. Hydroxyproline in the supernatants was measured twice by colorimetric determination using a Hydroxyproline Assay Kit (Sigma-Aldrich).

### Liver Histopathology and Immunohistochemistry for Pre- and Posttreatment Paired Liver Biopsies

Pre- and posttreatment liver biopsies were formaldehyde-fixed, paraffin-embedded, and stained with H&E and PSR, and scored in a blinded manner by a pathologist according to the criteria developed by Kleiner et al. (19) The liver slides were evaluated for steatosis (0–3), inflammation (0–3), ballooning (0–2), the nonalcoholic fatty liver disease (NAFLD) activity score (NAS, sum of individual components, 0–8), and fibrosis stage (stage 0–4). Immunohistochemistry (IHC) staining of Col1a1 (Southern Biotech, Cat. No. 1310-01, 1:100 dilution), α-smooth muscle actin (α-SMA, Abcam, Cat. No. Ab124964, 1:800 dilution), collagen IV (Col IV, Southern Biotech, Cat. No. 1340-01, 1:100 dilution), or laminin (Dako, Cat. No. Z0097, 1:800 dilution) for fibrosis, and Galectin-3 for fibrosis/inflammation (Biolegend, Cat. No. 125402, 1:50,000 dilution) was performed according to the manufacturer’s instructions. Stained liver slides were quantitatively assessed using digital imaging software Visiomorph (Visiopharm) and expressed as the fractional area (%) relative to total tissue area. For quantitative assessment of fibrosis and inflammation, PSR- and IHC-stained area was expressed as fractional area (%) relative to total tissue area after subtracting corresponding fat area by steatosis image analysis.

### Heavy Water Labeling and Determination of Hepatic Collagen Fractional Synthesis Rate

Hepatic new collagen synthesis was measured from its fractional synthesis rate (FSR). Mice were given a priming dose of 100% heavy water (D_2_O) in 0.9% NaCl by intraperitoneal injection to immediately set the body water enrichment level to ∼5%. This level was then maintained by providing the mice with 8% D_2_O in the daily drinking water for the last 7 days before euthanasia. The detailed methods for heavy water labeling and hepatic collagen synthesis measurement in vivo were described previously (22).

### RNA Transcriptomics

Purified RNA (10 ng–1 μg) from terminal liver (*n* = 8 per group) was used to generate a single cDNA library for each mouse using the NEBNext Ultra II Directional RNA Library Prep Kit for Illumina (New England BioLabs, Inc., Ipswich, MA). The cDNA libraries were then sequenced on a NextSeq 500 using NextSeq 500/550 High Output Kit V2 (Illumina, San Diego, CA). The sequencing data were aligned to the genome of the GRCm38 v89 Ensembl *Mus musculus* obtained from the Ensembl database using the Spliced Transcripts Alignment to a Reference (STAR) software with default parameters. For the bioinformatic analysis, the quality of the data was evaluated using the standard RNA-sequencing quality control parameters, the inter- and intragroup variability was evaluated using principal component analysis (PCA) using R package FactoMineR and hierarchical clustering, and the differentially expressed genes were identified using the R-package DESeq2. RNAseq data were presented as Reads Per Kilobase per Million mapped reads (RPKM).

### Statistical Analyses

JMP statistical software (version 17; SAS Institute, Cary, NC) was used for statistical analysis. Graphs were created in PRISM (v. 9; GraphPad, San Diego, CA). Data were presented as means ± SE. One-way analysis of variance (ANOVA) followed by a post hoc Student’s *t* test was used to evaluate the difference between treatment groups. Statistically significant differences between treatment groups are presented using different letters; whenever treatment groups do not have a common letter (a, b, c, d, e, or f), they are statistically different (*P* value < 0.05). Pre- and posttreatment NAFLD activity score (NAS) comparison with the seladelpar group was made by analysis of covariance (ANCOVA) using treatment as a factor and prebiopsy NAS as a covariate. Comparison with the seladelpar group for a decrease of NAS by ≥ 2 points was performed using Fisher’s exact test.

The RNAseq data in this study have been deposited in the NCBI sequence read archives (SRA) under BioProject accession number PRJNA1032138.

## RESULTS

### Seladelpar Alone and in Combination with Liraglutide or Selonsertib Improved Liver Enzymes and Lipids

**Figure 1.**
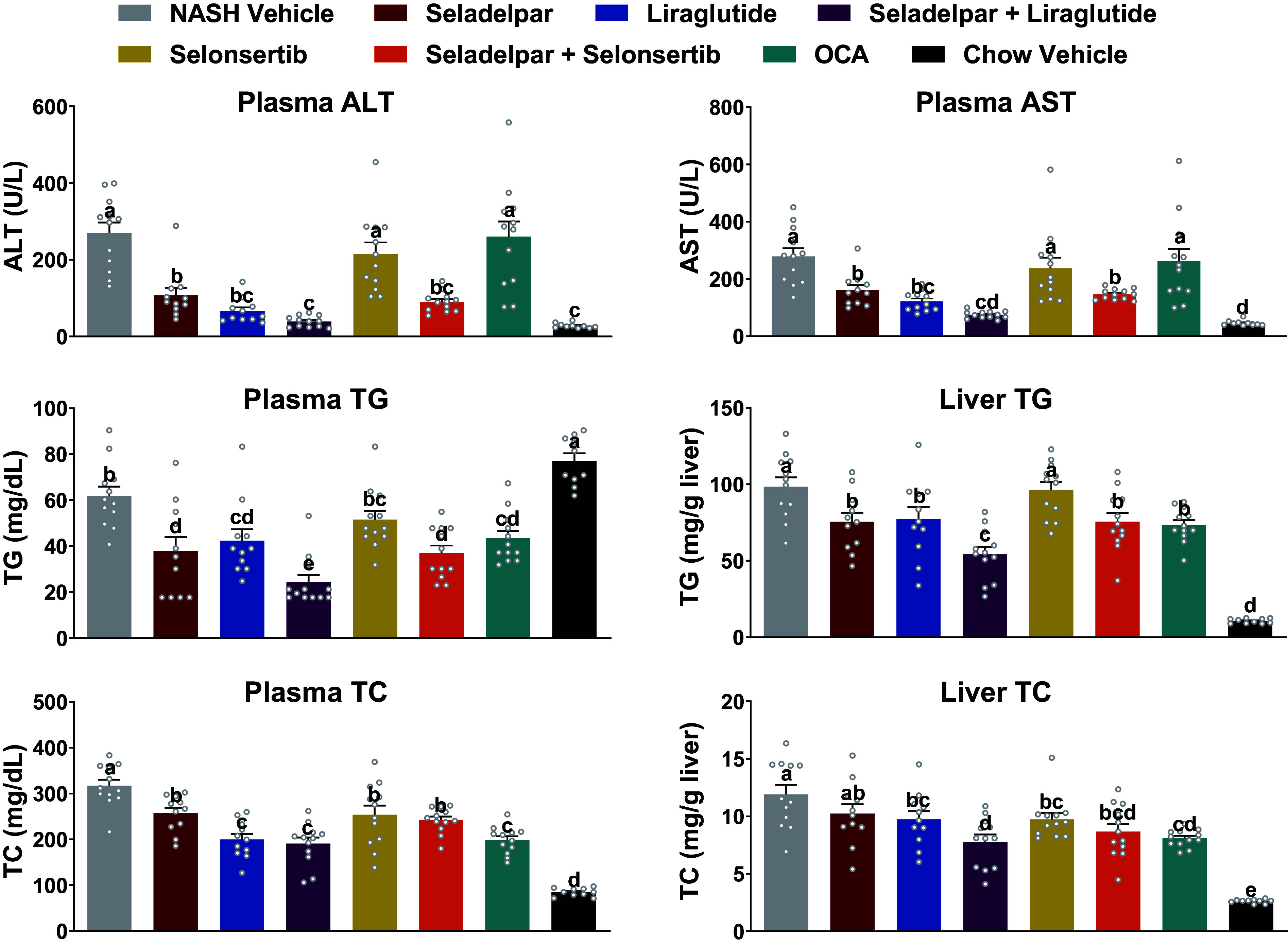
Effect of seladelpar, liraglutide, selonsertib, OCA, or seladelpar combination with liraglutide or selonsertib on ALT, AST, TG, and TC. Data are presented as means ± SE (*n* = 9–12 mice per group). Statistically significant differences between treatment groups are presented using different letters; whenever treatment groups do not have a common letter (a, b, c, d, or e), they are statistically different (*P* value < 0.05). ALT, alanine aminotransferase; AST, aspartate aminotransferase; OCA, obeticholic acid; TC, total cholesterol; TG, triglyceride.

Seladelpar and liraglutide alone or seladelpar in combination with liraglutide or selonsertib resulted in significant reductions in plasma ALT and AST levels compared with the NASH Vehicle. In contrast, neither selonsertib nor OCA alone had significant effects on ALT or AST levels. The seladelpar and liraglutide combination led to further reductions in ALT and AST to levels that were similar to those of the normal Chow Vehicle (Fig. 1). All monotherapies except selonsertib resulted in similar significant levels of reductions in plasma and liver triglycerides (TG) levels compared with the NASH Vehicle. A combination of seladelpar and liraglutide achieved a significant and additional reduction compared with monotherapies in plasma and liver TG levels. Several mono- and combination therapies reduced plasma and liver total cholesterol (TC) compared with NASH Vehicle (Fig. 1). During the 12 wk of the treatment period, NASH Vehicle or Chow Vehicle group mice maintained steady body weight (Supplemental Fig. S2*A*). Consistent with its clinically observed effect on weight loss (23), liraglutide resulted in 11% weight loss relative to NASH Vehicle after 12 wk. Treatment with seladelpar alone resulted in 9% weight loss, whereas combination treatment resulted in a nearly additive reduction in weight (18%). These effects on weight occurred in the absence of obvious differences in food intake among groups, although liraglutide and its combination with seladelpar did cause an expected initial sharp reduction in food intake that returned to near that of the NASH Vehicle over time (Supplemental Fig. S2*B*). Liraglutide treatment resulted in substantial reduction in liver weight. Although seladelpar caused a small, nonsignificant increase in liver weight relative to NASH Vehicle, the combination of seladelpar and liraglutide resulted in a reduction in liver weight. Selonsertib and OCA caused a small decrease in liver weight. Similar results were observed with liver-to-body weight ratio (Supplemental Fig. S2, *C* and *D*).

### Seladelpar Alone or in Combination with Other Agents Resulted in Stark Reductions in Liver Fibrosis and New Collagen Synthesis Rate

**Figure 2.**
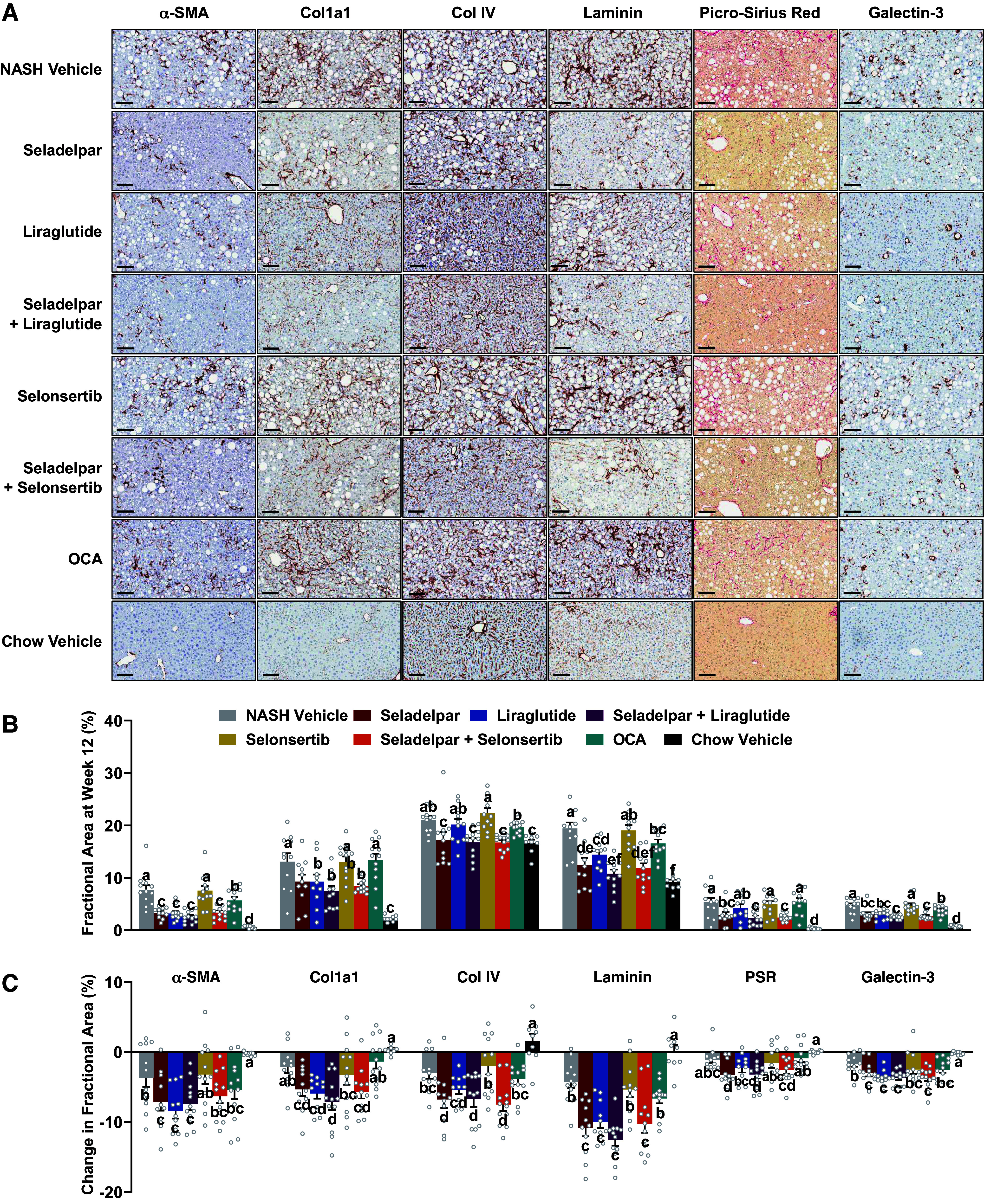
Mono- and combination treatments of seladelpar with liraglutide or selonsertib improve liver histopathology in DIO-NASH mice. *A*: representative posttreatment liver sections stained with α-SMA, Col1a1, Col IV, laminin, Picro-Sirius Red (PSR) for fibrosis, and Galectin-3 for fibrosis/inflammation (magnification ×20, scale bar = 100 µm). Quantitative morphometric analysis of posttreatment fractional area (%, *top, B*) and changes in the fractional area from baseline (%, *bottom, C*). Substantial decreases of fibrosis (α-SMA, Col1a1, Col IV, laminin, and PSR) and fibrosis/inflammation (Galectin-3) were observed in mono- and combination of seladelpar with liraglutide or selonsertib. Data for continuous variables are presented as means ± SE (*n* = 9–12 mice per group). Statistically significant differences between treatment groups are presented using different letters; whenever treatment groups do not have a common letter (a, b, c, d, e, or f), they are statistically different (*P* value < 0.05). DIO-NASH, diet-induced obese NASH; NASH, nonalcoholic steatohepatitis.

Diet-induced obese NASH (DIO-NASH) mice with at least stage 1 fibrosis before treatment developed marked liver fibrosis by the end of the treatment period. Fibrosis in the liver was visualized by collagen deposition using Picro-Sirius Red (PSR) staining and immunohistochemistry (IHC) of fibrosis markers (Fig. 2*A*). Substantial reductions in fibrosis were observed in the seladelpar treatment groups compared with NASH Vehicle. Figure 2*B* shows the quantitative morphometric assessment of these markers. At baseline, the amylin liver NASH (AMLN) diet resulted in a mean 15% of the fractional area of Col1a1 immunostaining in DIO-NASH mouse cohort compared with the 2% observed in Chow-fed mice. The fractional areas of PSR, α-SMA, and Col1a1 staining were significantly reduced by seladelpar alone or in combination with liraglutide or selonsertib after 12 wk of treatment. Changes in fractional area were measured by immunostaining of paired biopsies of pre- and posttreatment sections (Fig. 2*C*). Significant reductions in the fibrotic area from baseline were observed with seladelpar and liraglutide alone and in seladelpar combination groups. Collagen IV and laminin are major components of basement membranes that serve as markers of the perisinusoidal basement membrane formation that are associated with the severity of liver fibrosis (24). Both Collagen IV and laminin fractional staining areas were significantly lowered by treatment with seladelpar as monotherapy and in combination with liraglutide or selonsertib (Fig. 2*C*). OCA treatment did not result in significant changes in the pre- and poststaining area for any of these markers.

**Figure 3.**
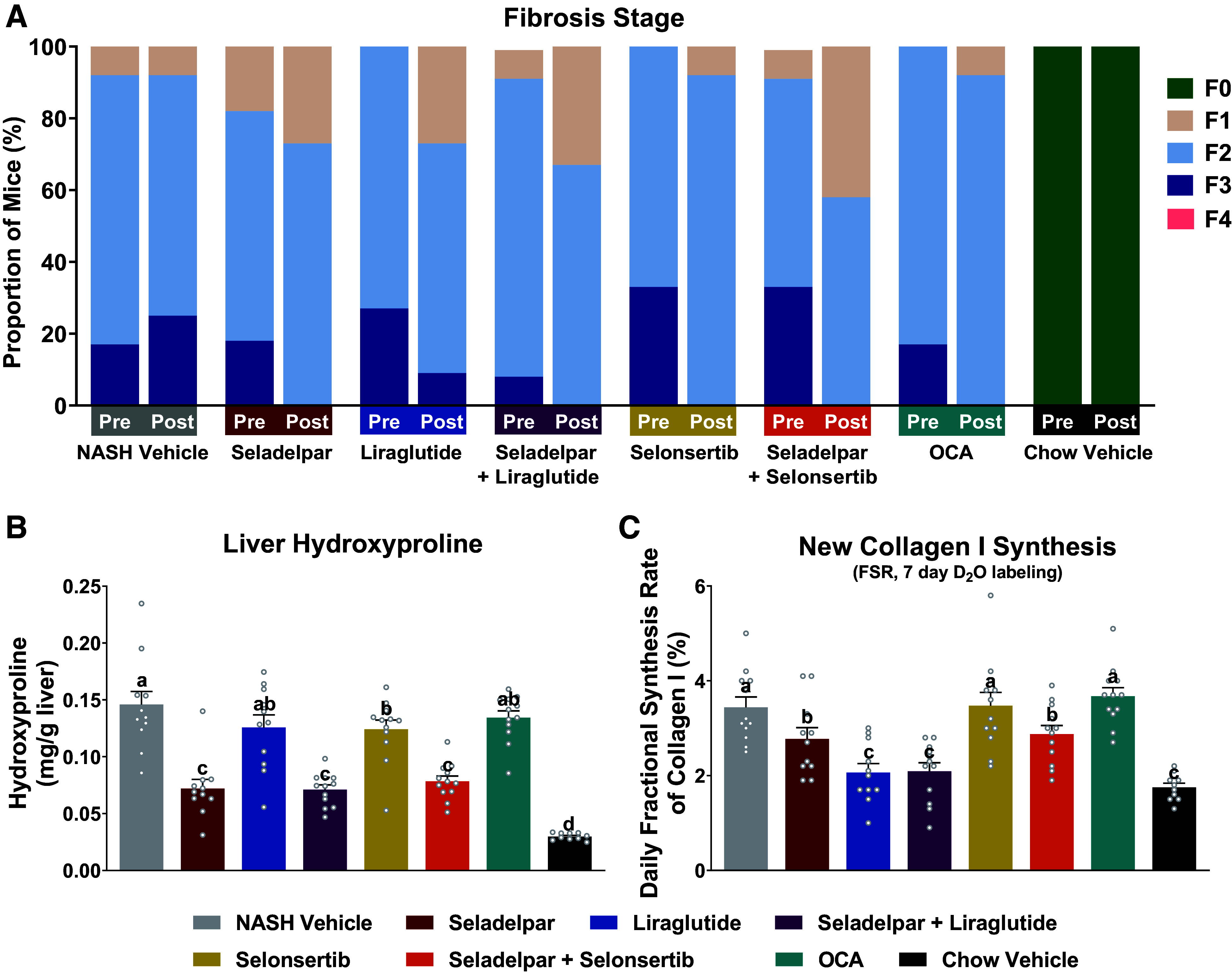
Mono- and combination treatments of seladelpar with liraglutide or selonsertib improve liver fibrosis in DIO-NASH mice. *A*: fibrosis stage. *B*: hepatic hydroxyproline content. *C*: new collagen I synthesis rate. Data for continuous variables are presented as means ± SE (*n* = 9–12 mice per group). Statistically significant differences between treatment groups are presented using different letters; whenever treatment groups do not have a common letter (a, b, c, or d), they are statistically different (*P* value < 0.05). DIO-NASH, diet-induced obese NASH; NASH, nonalcoholic steatohepatitis.

At baseline, the distribution of fibrosis stages for the DIO-NASH mouse cohort was 6% (stage 1), 72% (stage 2), and 22% (stage 3), respectively. None of the mice had developed fibrosis stage 4 at baseline or during the treatment period. No improvement in fibrosis stage was noted in the NASH Vehicle group and 92% of the mice remained at the same fibrosis stage as at baseline. In monotherapy, improvements in fibrosis stage (≥ 1) were observed in mice treated with OCA (25%), seladelpar (27%), selonsertib (42%), and liraglutide (45%). The most substantial improvement in fibrosis stage was noted in the seladelpar and selonsertib combination group (67%), whereas the combination of seladelpar and liraglutide displayed a marginal improvement (33%) compared with seladelpar alone. Stage 3 fibrosis was noted in all treatment groups at the start of treatment. However, all active treatment groups, except for liraglutide monotherapy, abolished stage 3 fibrosis at the end of the treatment period (Fig. 3*A*).

Although histopathology (Fig. 3*A*) demonstrated the efficacy of each of the treatments used in the study, we extended the studies to include quantitative measures of different components reflecting fibrosis. These results demonstrated clear differences between the treatments. The effects of seladelpar on fibrosis were further confirmed by assessing hydroxyproline content, which reflects the total amount of collagen in the liver (Fig. 3*B*). A substantial reduction of hydroxyproline content (∼50% of NASH Vehicle) was only observed in the seladelpar-treated groups (alone and in combination with other agents). Thus, it appeared that seladelpar contributed to the reduction of fibrosis in combination groups by its unique impact on collagen detected as hydroxyproline. Liraglutide, selonsertib, and OCA alone decreased hydroxyproline content by 14%, 15%, and 8% relative to NASH Vehicle, respectively, without a statistical difference among these three groups. Moreover, a significant decrease in the daily fractional synthesis rate (FSR) of new collagen I protein (from D_2_O labeling and tandem mass spectrometric analysis) was observed in seladelpar, liraglutide alone, and seladelpar in combination with liraglutide or selonsertib. New collagen I protein FSR in liraglutide alone and in combination with seladelpar resulted in a similar FSR as in Chow-fed mice (Fig. 3*C*). Seladelpar decreased both established liver fibrosis as well as new collagen synthesis rate, whereas liraglutide decreased new collagen synthesis rate without any significant effect on the resolution of established fibrosis. Although OCA improved fibrosis stage, it had no significant effects on either liver hydroxyproline or new collagen synthesis compared with NASH Vehicle.

### Seladelpar and Its Combination with Liraglutide or Selonsertib Improved Hepatic Steatosis and NAFLD Activity Score

**Figure 4.**
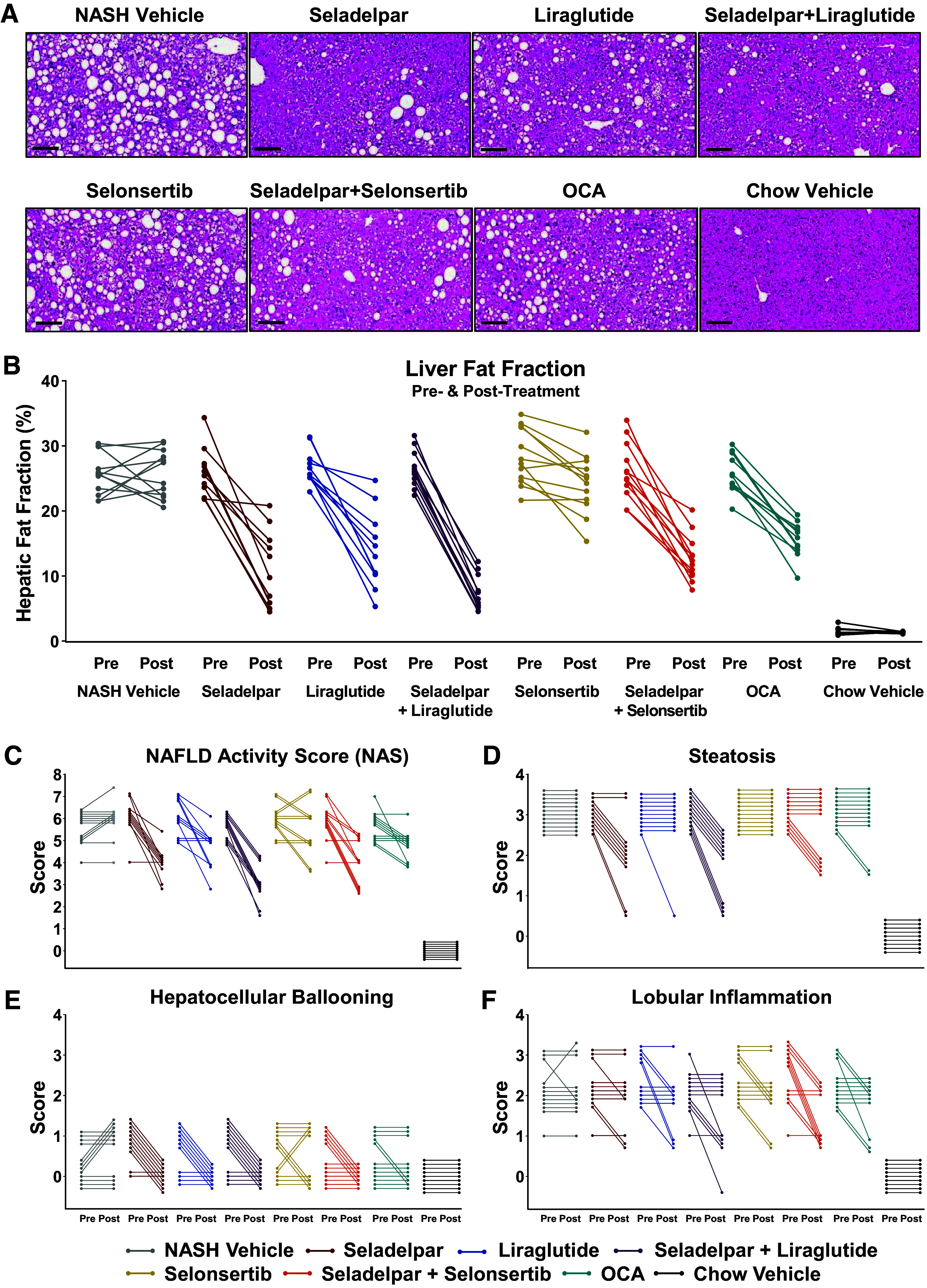
Mono- and combination treatments of seladelpar with liraglutide or selonsertib improve hepatic steatosis, NAFLD activity score (NAS), and its components. *A*: representative posttreatment liver sections stained with hematoxylin-eosin for steatosis (magnification ×20, scale bar = 100 µm). Changes in the individual liver fat fraction (%) (*B*), NAFLD activity score (*C*), steatosis (*D*), hepatocellular ballooning (*E*), and lobular inflammation (*F*). NAFLD, nonalcoholic fatty liver disease.

The mean baseline fractional liver fat was 26% in the DIO-NASH mouse cohort compared with 1.3% in Chow Vehicle (Fig. 4, *A* and *B*). Significant and substantial reductions in liver steatosis were observed in seladelpar and liraglutide combination (76%) > seladelpar (57%) > seladelpar and selonsertib combination (51%) > liraglutide (47%) > OCA (38%) while the reduction observed with selonsertib (15%) did not reach statistical significance compared with NASH Vehicle (Supplemental Fig. S3*A*).

The NAS and its components for individual mice are shown in Fig. 4, *C–F*. The mean NAS at baseline was 5.8 in the DIO-NASH mouse cohort (Fig. 4*C*). The proportion of mice with a two-point or greater improvement in NAS was 82% (seladelpar), 100% (seladelpar-liraglutide combination), 67% (seladelpar-selonsertib combination), 36% (liraglutide), 17% (selonsertib), 8% (OCA), and 0% (NASH Vehicle; Supplemental Fig. S3*B*). Greater reductions in steatosis score were seen in seladelpar alone (82%) and the seladelpar combination with liraglutide (100%) or selonsertib (42%) than other monotherapies OCA (17%), liraglutide (9%), and selonsertib (0%), respectively (Fig. 4*D*). Hepatocellular ballooning is a key feature of the diagnosis of NASH. At the beginning of the treatment, hepatocellular ballooning was observed in 55% of the DIO-NASH mouse cohort (Fig. 4*E*). Seladelpar, liraglutide, and the combinations that included seladelpar eliminated hepatocellular ballooning from the liver, whereas hepatocellular ballooning was persistent in the livers treated with NASH Vehicle, selonsertib, and OCA alone. When compared with NASH Vehicle, a lowering of Galectin-3, an inflammatory marker, was observed in seladelpar, liraglutide, and seladelpar combinations (Fig. 2, *A–C*). Reductions in lobular inflammation score were noted in some of the mice in all active treatment groups (Fig. 4*F*). For seladelpar and its combinations, overall improvements in the NAS were attributed to the reductions in all its components: steatosis, hepatocellular ballooning, and lobular inflammation (Fig. 4, *C–F*).

### Liver Transcriptomic Analysis Reveals Complementary Effects of Combined Therapeutics

Mice fed on the AMLN diet over 1 year resulted in dramatic changes in gene expression compared with Chow-fed mice (Supplemental Fig. S4). Volcano plots allowed visualization of overall changes in gene expression in treatment groups compared with NASH Vehicle.

**Figure 5.**
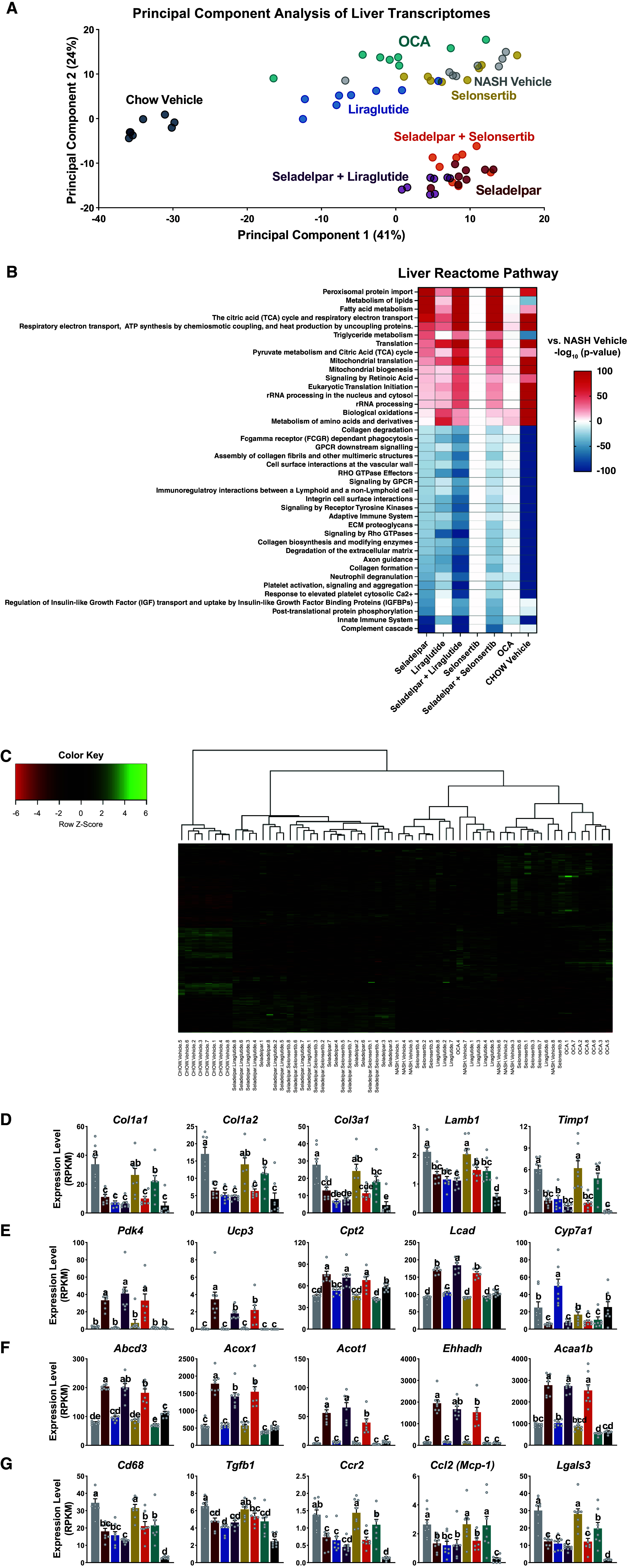
Substantial changes in hepatic gene expression by seladelpar, liraglutide, and its combination with liraglutide or selonsertib. *A*: principal component analysis (PCA) of the 500 most variable gene expression levels. *B*: overview of liver REACTOME pathway enrichment analysis indicates the pathways that were significantly regulated by treatment compared with NASH Vehicle. Red indicates upregulation and blue indicates downregulation. *C*: clustered heatmaps of the 500 most variable genes differentially expressed. Green color represents a higher and the red color represents lower expression level. Black color indicates no differential expression. The expression profiles of individual mice were clustered based on their transcriptional similarities. Genes were analyzed for their reported association with fibrosis (*D*), lipid metabolism (*E*), peroxisomal fatty acid β-oxidation (*F*), and inflammation (*G*). Data for continuous variables are presented as means ± SE (*n* = 8 mice per group). Statistically significant differences between treatment groups are presented using different letters; whenever treatment groups do not have a common letter (a, b, c, d, or e), they are statistically different (*P* value < 0.05). NASH, nonalcoholic steatohepatitis; OCA, obeticholic acid.

Seladelpar treatment significantly up- or downregulated a large number of genes. The pattern of changes in gene expression observed was similar in seladelpar monotherapy and when administered with other treatments (Fig. 5). In a principal component analysis (PCA) using the 500 most variable genes, distinct patterns of hepatic gene expression were shown by the treatment groups (Fig. 5*A*). A clear separation between Chow Vehicle and DIO-NASH cohort was observed. Among the DIO-NASH cohort, liraglutide, OCA, and selonsertib treatment groups had partially overlapping transcriptional patterns with those of NASH Vehicle. Seladelpar treatment resulted in a unique gene expression pattern compared with other monotherapies indicating the diversity of pathways regulated by each agent. Seladelpar in combination with liraglutide or selonsertib segregated near seladelpar monotherapy and shared similar features of gene expression indicating that changes in gene expression in the seladelpar combination were predominantly a result of the effects of seladelpar (Fig. 5*B*). Peroxisomal protein import, metabolism of lipids, and fatty acid metabolism were prominently increased by seladelpar as expected for a PPARδ agonist. Liraglutide alone had little impact on these pathways. Liraglutide increased expression of components of the liver reactome associated with translation, biological oxidations, and metabolism of amino acids and derivatives that were not associated with seladelpar treatment. Selonsertib-treated livers displayed little change in RNA expression from the NASH Vehicle livers, and OCA-treated livers displayed smaller elevations of components of respiratory electron transport and ATP synthesis, biological oxidations, and metabolism of amino acids and derivatives. For pathway groups that were decreased by compound treatment, seladelpar had its largest impact on innate immunity and complement cascade pathways. Liraglutide had minimal effects on these pathways. The pattern of activities of seladelpar and liraglutide in this pathway analysis was overlapping but clearly distinct. The set of pathway changes associated with the combination of seladelpar and liraglutide appeared to be additive.

The clustering of mice and the similarity of their gene expression patterns were displayed in Fig. 5*C*. The clustering confirmed that seladelpar and its combination groups share similar gene expression patterns.

Examining the individual gene expression levels across all treatments, there were marked reductions in major mRNA markers of fibrosis, including *Col1a1, Col1a2, Col3a1, Lamb1*, and *Timp1* with seladelpar, liraglutide, and the combinations with the former (Fig. 5*D*). These changes were consistent with the reductions in fibrosis with these treatments but did not explain the superior ability of seladelpar to reduce hydroxyproline content in the liver. Thus, the fibrosis-resolving properties of seladelpar may be due to its unique effect on components of metabolism and lipid metabolism in particular. *Pdk4* expression was increased solely in mice treated with seladelpar and its combinations (Fig. 5*E*). This contributes to the switch of the fuel for hepatic energy metabolism from glucose to fatty acid β-oxidation (25). The mitochondrial uncoupling protein *Ucp3*, the mitochondrial inner membrane fatty acyl-carnitine transporter *Cpt2*, and the mitochondrial fatty acid β-oxidation enzyme *Lcad* are among the multiple genes involved in lipid metabolism that are uniquely upregulated by treatments that include seladelpar whether alone or in combination with agents with differing mechanisms. Similar to OCA, seladelpar and its combination significantly reduced *Cyp7a1*. Interestingly, liraglutide caused an increase in *Cyp7a1* in this mouse model, but the combination liraglutide and seladelpar resulted in a decrease similar to seladelpar alone (Fig. 5*E*). Peroxisomal enzymes involved in fatty acid β-oxidation were also robustly induced by seladelpar (Fig. 5*F*). These large changes in expression suggest a major reorganization of metabolism by seladelpar.

Excess lipid in hepatocytes leads to accumulation of toxic intermediates that trigger inflammatory processes that also initiate fibrosis (26). In the NASH mouse model, the AMLN diet increased expression of the inflammatory mRNA markers *Cd68, Tgfb1, Ccr2, Ccl2* (*Mcp-1*), and *Lgals3* among others (Fig. 5*G*). Both seladelpar and liraglutide substantially reduced the expression of these genes reflecting an overall decrease in inflammatory signaling in the liver. These results are consistent with previous studies in mice for both seladelpar (11) and liraglutide (27). Notably, OCA and selonsertib demonstrated smaller reductions in these inflammatory markers in this study.

## DISCUSSION

Liver fibrosis is the excessive deposition of extracellular matrix (ECM) proteins, including collagen, due to chronic inflammation or liver injury caused by viral infection, alcoholic or nonalcoholic steatohepatitis, metabolic, autoimmune, or cholestatic insult (28, 29). Kupffer cells and activated hepatic stellate cells are leading participants in the initiation and progression of liver fibrosis as well as its resolution (26). Advanced fibrosis leads to loss of essential liver function that can ultimately lead to mortality or the need for transplantation. As single agents have demonstrated limited ability to reverse fibrotic changes in clinical studies, in the present study, we compared treatment effects of monotherapy (seladelpar, liraglutide, selonsertib, or OCA) and combinations of seladelpar with liraglutide or selonsertib on fibrosis, fibrogenesis, and NASH histopathology in an AMLN diet-induced mouse model using paired liver biopsy at baseline and study termination. This before and after liver fibrosis assessment is critical for sensitive evaluation of compound efficacy. We demonstrated that four therapeutic agents that work by distinct but overlapping pathways each improve liver fibrosis and NASH significantly in a DIO-NASH mouse model and that combinations of such agents can be effective in concert.

The selective PPARδ agonist seladelpar regulates the transcription of numerous genes in multiple tissues including in the liver. PPARδ is known to play an important role in the regulation of glucose and lipid metabolism, energy expenditure, and inflammation (30–34). In this study, seladelpar demonstrated a robust reduction in liver fibrosis as evidenced by significant reductions in *1*) preexisting total collagen as measured by hydroxyproline content (nearly 50%), *2*) new collagen I synthesis rate, *3*) expression of genes encoding collagens and basement membrane proteins (*Col1a1, Col1a2, Col3a1*, and *Lamb1*), and tissue inhibitor of matrix metalloproteinases (*Timp1*), *4*) area stained with fibrosis markers (PSR, α-SMA, Col1a1, Col IV, laminin, and Galectin-3), and *5*) the proportion of mice with higher stages of fibrosis. The results reported here are consistent with a report in diabetic obese *foz/foz* mice that seladelpar independent of weight loss demonstrated regression of atherogenic diet-induced fibrosis by PSR staining as well as lower mRNA levels of *α-Sma*, *Col1a1*, connective tissue growth factor (*Ctgf*), and *Timp1* mRNA (11).

As hepatocytes, Kupffer cells, and hepatic stellate cells do not express the GLP-1 receptor, it is more likely that GLP-1 agonists act on fibrosis via indirect actions rather than direct liver engagement (35, 36). Nevertheless, in this study, the GLP-1RA liraglutide reduced mRNA levels of fibrosis markers (*Col1a1, Col1a2, Col3a1, Lamb1*, and *Timp1*) and the synthesis rate of collagen I but showed mixed responses in staining of fibrosis markers: decreases in α-SMA and Col1a1 with no significant changes in Col IV and PSR were observed compared with NASH Vehicle. There was no significant reduction in hydroxyproline content with liraglutide compared with NASH Vehicle. In other preclinical studies, liraglutide treatment was found to result in a significant reduction in mRNA levels of fibrosis markers without any histological improvement in fibrosis (36, 37). Both selonsertib and OCA showed improvements in fibrosis stage without changes in any of the fibrosis marker mRNAs or by staining for fibrosis markers, hydroxyproline content, or new collagen I synthesis.

A substantial reduction in hepatic fat was seen in seladelpar, liraglutide, OCA, and seladelpar combination groups. Both liraglutide and seladelpar resulted in weight loss without significant changes in food intake. Liraglutide acts via GLP-1 receptor to cause weight loss through effects on pancreatic islet hormones and likely via fibroblast growth factor 21 (FGF21) production from the liver in mice (38). Recent studies demonstrated that seladelpar directly increases FGF21 production in the liver of mice via transcriptional regulation (39). Seladelpar increases endogenous liver production of FGF21, which may have advantages over peripheral administration of exogenous FGF21 analogs. FGF21 is known to regulate body weight in both mice and humans although the impact of this factor is somewhat different in the two species due to a fibroblast activation protein (FAP) cleavage site in the latter that leads to inactivation of the protein (40, 41). A stabilized form of FGF21 effectively reduced NASH pathology, including fibrosis, in phase 2 clinical studies (42, 43). In part, the decrease in NASH pathology observed in the current study may be due to changes in liver-derived FGF21. Therefore, the combination of seladelpar and liraglutide may provide alternative pathways for raising this member of the FGF21 family beyond what each agent alone can achieve.

FXR agents such as OCA impact NASH pathology in mice via induction of FGF15, a human FGF19 ortholog, which acts on the liver to reduce the production of bile acids (44, 45). A modified form of FGF19 has also shown efficacy for improvement of NASH pathology in humans (46). FXR agonists also act directly on the liver by increasing the expression of SHP (43). Seladelpar can also reduce bile acid production by downregulation of its rate-limiting enzyme, *CYP7A1* (47). Again, activation of overlapping pathways such as these suggests combination of seladelpar and FXR agonists could show effects greater than each agent alone.

Enhancing confidence that these results for seladelpar in mice are relevant to human disease is the concordance of the gene expression changes brought about by this agent in mice with genome-wide association studies (GWAS) of human NAFLD and NASH. Not surprisingly given the importance of metabolism in the etiology of the disease, the majority of identified genetic polymorphisms in NAFLD impact lipid pathways (15). The GWAS signal for *PNPLA3* is strongly associated with NAFLD and also with a NASH cohort (48) and variants in *PLIN2, KLF6, UCP3*, and *MARC1* (49) are associated with liver steatosis, fibrosis, cirrhosis, or NAFLD. The regulation of these genes by seladelpar and its combinations with liraglutide or selonsertib in mouse liver is concordant with their expected biochemical or regulatory functions. The striking additivity of the combination of seladelpar and liraglutide relative to each agent alone in the liver reactome analysis of the RNAseq data suggests that these agents arrive at efficacy by impacting diverse components, and that their combination should be explored in clinical studies. The conformity of mouse RNAseq data and human genetics for NAFLD and NASH can guide future studies to enhance translational efficiency.

Liraglutide and seladelpar stood out in this study as the agents that best reduced liver injury in the face of a NASH diet as judged by serum ALT levels. They reduced inflammatory markers both individually and together. When combined, the two agents brought serum ALT down to those found with the Chow diet. As the high-fat diet was continued during treatment with both agents (and the combination of the two), both liraglutide and seladelpar appear to protect liver components from the deleterious effects of the excess lipid. For liraglutide, this may be due to metabolic and anti-inflammatory effects.

Recent metabolomics data for seladelpar provided evidence that increased peroxisomal and mitochondrial oxidation of lipids and increased turnover of acyl-carnitines led to a reduction in concentrations of toxic lipids (C. Y.-J, Johnson J, unpublished observations). Given these unique actions of seladelpar to protect liver function in the face of residual insults, future clinical trials in NASH and other liver diseases should consider combining this selective PPARδ agonist with other agents to reach the desired endpoint of preventing or delaying the need for liver transplantation.

### Perspectives and Significance

Studies elucidating NASH pathology have suggested that numerous genetic variations and metabolic changes, as well as alterations in the microbiome, contribute to disease progression. Furthermore, clinical trials have indicated that no single treatment sufficiently changes the course of NASH. Our study identified a particular combination of therapeutics with different mechanisms that improved efficacy in NASH relative to either alone. Further model studies in this vein could lead to efficient selection of combinations of methods for successful clinical engagement with this increasingly prevalent and often severe disease.

## DATA AVAILABILITY

Data will be made available upon reasonable request.

## SUPPLEMENTAL DATA

10.6084/m9.figshare.24451993 Supplemental Figs. S1–S4: https://doi.org/10.6084/m9.figshare.24451993.

## GRANTS

This study was funded by CymaBay Therapeutics, Inc.

## DISCLOSURES

Y-J.C., J-J.L., J.S., and C.A.M. are employees of CymaBay Therapeutics, Inc. J.D.J. and M.K.H. consult for CymaBay Therapeutics, Inc. None of the other authors has any conflicts of interest, financial or otherwise, to disclose.

## AUTHOR CONTRIBUTIONS

Y-J.C., J.D.J., M.K.H., and C.A.M. conceived and designed research; Y-J.C., J.S., M.M., and M.K.H. performed experiments; Y-J.C., J.D.J., J-J.L., and M.K.H. analyzed data; Y-J.C., J.D.J., M.K.H., and C.A.M. interpreted results of experiments; Y-J.C. and J-J.L. prepared figures; Y-J.C. and J.D.J. drafted manuscript; Y-J.C., J.D.J., M.K.H., and C.A.M. edited and revised manuscript; Y-J.C., J.D.J., J-J.L., J.S., M.M., M.K.H., and C.A.M. approved final version of manuscript.
